# The Early Growth Genetics (EGG) and EArly Genetics and Lifecourse Epidemiology (EAGLE) consortia: design, results and future prospects

**DOI:** 10.1007/s10654-019-00502-9

**Published:** 2019-03-18

**Authors:** Christel M. Middeldorp, Janine F. Felix, Anubha Mahajan, EArly Genetics Lifecourse Epidemiology (EAGLE) consortium, Tarunveer S. Ahluwalia, Juha Auvinen, Meike Bartels, Jose Ramon Bilbao, Hans Bisgaard, Klaus Bønnelykke, Dorret I. Boomsma, Jonathan P. Bradfield, Mariona Bustamante, Zhanghua Chen, John A. Curtin, Adnan Custovic, George Davey Smith, Gareth E. Davies, Liesbeth Duijts, Peter R. Eastwood, Anders U. Eliasen, Xavier Estivill, David M. Evans, Iryna O. Fedko, Janine F. Felix, W. James Gauderman, Frank Gilliland, Raquel Granell, Struan F. A. Grant, Monica Guxens, Hakon Hakonarson, Catharina A. Hartman, Joachim Heinrich, Anjali K. Henders, John Henderson, Patrick Holt, Jouke-Jan Hottenga, Elina Hyppönen, Carmen Iñíguez, Bo Jacobsson, Vincent W. V. Jaddoe, Marjo-Riitta Järvelin, Astanand Jugessur, Mika Kähönen, Jaakko Kaprio, Ville Karhunen, John P. Kemp, Gerard H. Koppelman, Ashish Kumar, Jari Lahti, Henrik Larsson, Debbie A. Lawlor, Terho Lehtimäki, Jin Li, Paul Lichtenstein, Sebastian Lundström, Leo-Pekka Lyytikäinen, Per Magnus, Abdullah A. Mamun, Minna Mannikko, Nicholas G. Martin, Hamdi Mbarek, Sarah E. Medland, Erik Melén, Christel M. Middeldorp, Jackob M. Najman, Michel G. Nivard, Ilja M. Nolte, Albertine J. Oldehinkel, Katja Pahkala, Teemu Palviainen, Lavinia Paternoster, Craig E. Pennell, Göran Pershagen, Niina Pitkänen, Robert Plomin, Beate St Pourcain, Christine Power, Lea Pulkkinen, Katri Räikkönen, Olli T. Raitakari, Rebecca C. Richmond, Fernando Rivadeneira, Richard J. Rose, Loreto Santa-Marina, James G. Scott, Sylvain Sebert, Saskia Selzam, Angela Simpson, Patrick M. A. Sleiman, Harold Snieder, Marie Standl, Camilla Stoltenberg, David P. Strachan, Leon Straker, Timo Strandberg, Jordi Sunyer, Elisabeth Thiering, Henning Tiemeier, Nicholas J. Timpson, Maties Torrent, André G. Uitterlinden, Toos van Beijsterveldt, Peter J. van der Most, Cornelia M. van Duijn, Jorma Viikari, Natalia Vilor-Tejedor, Judith M. Vonk, Tanja G. M. Vrijkotte, Eero Vuoksimaa, Carol A. Wang, Andrew J. O. Whitehouse, Gonneke Willemsen, Gail M. Williams, Naomi R. Wray, Shujing Xu, Cheng-Jian Xu, Lu Yi, Mohammad Hadi Zafarmand, Early Growth Genetics (EGG) consortium, Mark I. McCarthy

**Affiliations:** 1 Child Health Research Centre, University of Queensland, Brisbane, QLD Australia; 2 Child and Youth Mental Health Service, Children’s Health Queensland Hospital and Health Service, Brisbane, QLD Australia; 3 Department of Biological Psychology, Vrije Universiteit Amsterdam, 1081 BT Amsterdam, The Netherlands; 4 The Generation R Study Group, Erasmus MC, University Medical Center Rotterdam, 3015 CE Rotterdam, The Netherlands; 5 Department of Epidemiology, Erasmus MC, University Medical Center Rotterdam, 3015 CE Rotterdam, The Netherlands; 6 Department of Pediatrics, Erasmus MC, University Medical Center Rotterdam, 3015 CE Rotterdam, The Netherlands; 7 Wellcome Centre for Human Genetics, University of Oxford, Oxford, OX3 7BN UK; 8 Oxford Centre for Diabetes, Endocrinology and Metabolism, University of Oxford, Oxford, OX3 7LE UK; 9 Oxford National Institute for Health Research (NIHR) Biomedical Research Centre, Churchill Hospital, Oxford, OX3 7LE UK

**Keywords:** Genetics, Consortium, Childhood traits and disorders, Longitudinal

## Abstract

The impact of many unfavorable childhood traits or diseases, such as low birth weight and mental disorders, is not limited to childhood and adolescence, as they are also associated with poor outcomes in adulthood, such as cardiovascular disease. Insight into the genetic etiology of childhood and adolescent traits and disorders may therefore provide new perspectives, not only on how to improve wellbeing during childhood, but also how to prevent later adverse outcomes. To achieve the sample sizes required for genetic research, the Early Growth Genetics (EGG) and EArly Genetics and Lifecourse Epidemiology (EAGLE) consortia were established. The majority of the participating cohorts are longitudinal population-based samples, but other cohorts with data on early childhood phenotypes are also involved. Cohorts often have a broad focus and collect(ed) data on various somatic and psychiatric traits as well as environmental factors. Genetic variants have been successfully identified for multiple traits, for example, birth weight, atopic dermatitis, childhood BMI, allergic sensitization, and pubertal growth. Furthermore, the results have shown that genetic factors also partly underlie the association with adult traits. As sample sizes are still increasing, it is expected that future analyses will identify additional variants. This, in combination with the development of innovative statistical methods, will provide detailed insight on the mechanisms underlying the transition from childhood to adult disorders. Both consortia welcome new collaborations. Policies and contact details are available from the corresponding authors of this manuscript and/or the consortium websites.

## Background

In countries with a high-sociodemographic index, the major contributors to burden of disease during childhood and adolescence are non-communicable diseases such as obesity, asthma or allergies, and psychiatric disorders. These have a large cumulative impact on individuals, families and society [1]. Moreover, many early-life traits track throughout childhood and adolescence into adulthood. Childhood obesity, for example, is associated with adult obesity and cardiovascular disease [2]. Several childhood psychiatric disorders persist into adolescence and adulthood or precede severe mental illness such as schizophrenia, which usually starts at late adolescence or early adulthood [3, 4]. Low birth weight, as a proxy for a suboptimal intrauterine environment, has been shown to be robustly associated with many later-life non-communicable traits, including cardiovascular, respiratory and psychiatric disorders (see e.g., 5–7). This prompted researchers, including those within the Developmental Origins of Health and Disease (DOHaD) field, to investigate the basis for the early origins of later life differences in health and disease.

Insight into the etiology of childhood and adolescent traits and disorders may provide new perspectives, not only on how to improve wellbeing during childhood, but also how to prevent later adverse outcomes. Individual differences in developmental phenotypes, such as body weight and composition, behavioral problems, language skills, and their stability across ages are partly influenced by genetic factors [8–13]. Identifying the specific genetic variants that influence these traits, and the biological pathways through which they operate, can therefore help to unravel etiological mechanisms. Genetic studies can also define whether the relationships between childhood and adult traits, for example, birth weight and cardiovascular disease, are causally mediated by early life exposures. In addition, genetics can support how specific environmental factors contribute to variation in these traits, i.e., whether there is gene-environment interaction with the increase in risk depending on an individual’s genetic risk.

**Fig. 1:**
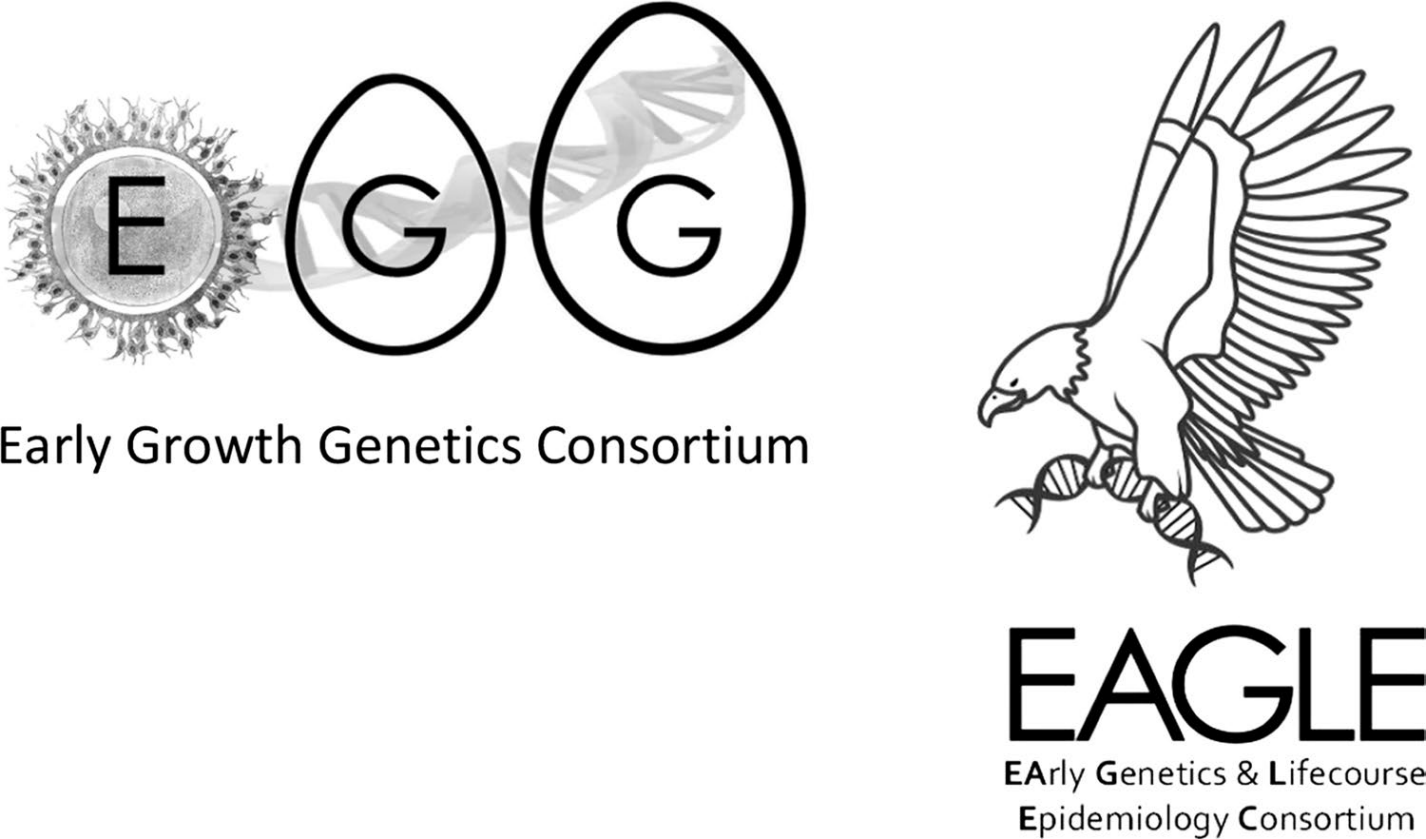
Logo’s

It is increasingly recognized that large sample sizes are essential in genetic research [14] and studies performed in large international consortia have become the norm. Two such consortia with a particular focus on the genetics of early life phenotypes are the Early Growth Genetics (EGG) consortium (http://egg-consortium.org/) and the EArly Genetics and Lifecourse Epidemiology (EAGLE) consortium (http://www.wikigenes.org/e/art/e/348.html) (Fig. 1). This paper describes these two consortia as they have shared objectives and the participating cohorts partly overlap. We also highlight the results so far and outline the directions of future research.

## Description and aims of the EGG and EAGLE consortia

Both consortia arose in 2009 out of the EU-funded European Network for Genetic And Genomic Epidemiology (ENGAGE). The EGG consortium focuses on the genetic basis of growth-related phenotypes spanning from fetal life into adolescence, including birth weight, childhood obesity and pubertal development. EAGLE was established to investigate the genetic basis of the wide range of further phenotypes collected by these cohorts from fetal life into adolescence, such as those relevant to asthma and eczema, childhood psychopathology, cognition, and neurodevelopment. The collective objectives of EGG and EAGLE are:

1. to characterize the genetic background of traits and diseases in fetal life, childhood and adolescence by facilitating collaboration between pregnancy, birth, childhood and adolescent cohort studies, as well as adult biobanks (such as UK Biobank) with relevant information;
2. to define the causal relationships between early life exposures and related early life phenotypes and major sources of morbidity and mortality in later life;
3. to develop and improve statistical methods for analyzing complex, high-dimensional and longitudinal phenotypic data;
4. to provide training opportunities for junior researchers to develop in the field of genetic epidemiology.

The EGG and EAGLE consortia started as collaborations of population-based pregnancy and birth cohort studies, each of which has collected longitudinal data across a wide range of developmental phenotypes. As the collaboration developed, cohorts that started data collection during childhood and adolescence were also included. Almost all participating studies have genome-wide genotype data available. In addition, early life data collected through self-report and/or record linkage in adult biobanks, such as UK Biobank or the population based cohorts listed in Table 1 that have an adult counterpart, have been brought into the genome-wide association (GWA) meta-analyses for phenotypes such as birth weight. Both consortia welcome new collaborations, and they are keen to add data from longitudinal cohorts that are currently in the process of obtaining genotype data.

**Table 1:** Participating cohorts

Short name	Full name cohort	Website	References
ABCD	Amsterdam Born Children and their Development	https://abcd-studie.nl/	20813863
ALSPAC	Avon Longitudinal Study on Parents and Children	www.bristol.ac.uk/alspac/	22507743, 22507742
B58C	1958 British Birth Cohort	www.cls.ioe.ac.uk/page.aspx?&sitesectionid=724&sitesectiontitle=Welcome+to+the+1958+National+Child+Development+Study	16155052, 17255346
BAMSE	Children, Allergy, Milieu, Stockholm, Epidemiology	https://ki.se/en/imm/bamse-project	26505741
BMDCS	Bone Mineral Density in Childhood Study	https://bmdcs.nichd.nih.gov/	17311856
Breathe	BRain dEvelopment and Air polluTion ultrafine particles in scHool childrEn	https://www.isglobal.org/en/-/breathe-brain-development-and-air-pollution-ultrafine-particles-in-school-children	25734425, 27656889
CATSS	Child and Adolescent Twin Study in Sweden	https://ki.se/en/meb/the-child-and-adolescent-twin-study-in-sweden-catss	22506305
CHOP	Children’s Hospital of Philadelphia	https://www.caglab.org/	22138692
CHS	Children’s Health Study	https://healthstudy.usc.edu/	10051249, 10051248, 17307103,25738666, 28103443, 27115265
CLHNS	Cebu Longitudinal Health and Nutrition Survey	http://www.cpc.unc.edu/projects/cebu	20507864
COPSAC	Copenhagen Prospective Studies on Asthma in Childhood	www.copsac.com	15521375, 24118234, 24241537
DNBC	Danish National Birth Cohort	https://www.ssi.dk/English/RandD/Research%20areas/Epidemiology/DNBC.aspx	
EFSOCH	Exeter Family Study of Childhood Health		16466435
Finntwin12	Finnish Twin Cohort Study	https://wiki.helsinki.fi/display/twineng/Twinstudy	23298696,17254406, 12537860
Gen3G	Genetics of Glucose regulation in Gestation and Growth	n/a	26842272
	Generation R Study	https://www.generationr.nl/	28070760; 25527369
GINIplus	German Infant Study on the influence of Nutrition Intervention PLUS environmental and genetic influences on allergy development	https://www.helmholtz-muenchen.de/epi/research/research-groups/allergy-epidemiology/projects/giniplus/index.html	20082618
GLAKU	Glycyrrhizin in Licorice	https://blogs.helsinki.fi/depsy-group/research/	19808634; 17076756; 11390327
HBCS	Helsinki Birth Cohort Study	https://thl.fi/en/web/thlfi-en/research-and-expertwork/projects-and-programmes/helsinki-birth-cohort-study-hbcs-idefix	11312225
Health2006	Helbred2006	https://clinicaltrials.gov/ct2/show/NCT00316667	23615486
INMA	INfancia y Medio Ambiente	http://proyectoinma.org/en_index.html	21471022
Inter99	The Inter99 Study	https://www.regionh.dk/rcph/population-based-epidemiology/Pages/The-Inter99-Study.aspx	14663300
LISA	Influence of life-style factors on the development of the immune system and allergies in East and West Germany	https://www.helmholtz-muenchen.de/epi/research/research-groups/allergy-epidemiology/projects/lisa/index.html	12358337
MAAS	Manchester Asthma and Allergy Study	http://maas.org.uk/	25805205, 15029579, 12688622
MOBA	Norwegian Mother and Child Cohort Study	https://fhi.no/studier/moba/	27063603,
MUSP	Mater University Study of Pregnancy	https://social-science.uq.edu.au/mater-university-queensland-study-pregnancy	25519422
NTR	Netherlands Twin Register	http://www.tweelingenregister.org/	23186620; 23265630
NFBC1966 and NFBC1986	Northern Finland Birth Cohort	http://www.oulu.fi/nfbc/	750195; 19060910; 9246691
PIAMA	Preventie en Incidentie van Astma en Mijt Allergie	http://piama.iras.uu.nl/	12688626, 23315435
	Project Viva	http://dacp.org/viva/	24639442
Qtwin	Queensland Twin Registry	http://www.qimrberghofer.edu.au/qtwin/	DOI: 10.1080/00049530410001734865
Raine	The Western Australian Pregnancy Cohort (Raine) Study	https://www.rainestudy.org.au/	8105165; 23230915; 23301674 l; 26169918; 28064197; 28662683
SKOT	Småbørns Kost Og Trivsel	https://skot.ku.dk/om-projektet/english/	28947836
STRIP	Special Turku Coronary Risk Factor Intervention Project	http://stripstudy.utu.fi/english.html	18430753
TCHAD	Twin Study of Child and Adolescent Development	https://ki.se/en/meb/twin-study-of-child-and-adolescent-development-tchad	17539366
TDCOB	The Danish Childhood Obesity Biobank	https://clinicaltrials.gov/ct2/show/NCT00928473	
TEDS	Twins Early Development Study	http://teds.ac.uk/	23110994
TRAILS	TRacking Adolescents’ Individual Lives Survey	https://www.trails.nl/	25431468
Young Finns	The Cardiovascular Risk in Young Finns Study	http://youngfinnsstudy.utu.fi/	18263651

Tables 1 and 2 provides a summary of the participating studies and their design, as of April 2018. Table 3 gives further details on the extensive data available, indicating, per cohort, whether data collection has taken place at least once at preschool, school, adolescent and adult age. However, many cohorts have had multiple follow-up rounds within any given period or follow-up data collection is ongoing, through research clinic assessments, questionnaires or record linkage. The majority of the cohorts have around equal numbers of males and females included.

**Table 2:** Study designs

Cohort	Study design	Years of recruitment	Country
ABCD	Population based pregnancy cohort	2003–2004	The Netherlands
ALSPAC	Population based birth cohort	1990–1992	UK
B58C	Population based birth cohort	1958	UK
BAMSE	Population based cohort	1994–1996	Sweden
BMDCS	Multi-center observational cohort	2002–2009	United States
Breathe	Population based cohort	2002–2006	Spain
CATSS	Populaton based twin birth cohort	1992-ongoing	Sweden
CHOP	Population based cohort	1988-Present	USA
CHS	Community based children cohort	1993–2002	United States
CLHNS	Population based birth cohort	1983–1984	Philippines
COPSAC-2000	Asthma risk birth cohort	From 2000-	Denmark
COPSAC-2010	Population based birth cohort	Ongoing From 2010	
COPSAC-REGISTRY	Severe asthma cases (children)	Ongoing	
DNBC-GOYA	Population based pregnancy cohorts	From 1997 Ongoing	Denmark
DNBC-PTB			
EFSOCH	Community-based pregnancy cohort of parent–offspring trios	2000–2004	United Kingdom
Finntwin12	Population-based twin-family cohort	1983–1987	Finland
Gen3G	Population based birth cohort	2010–2013	Canada
Generation R^a^	Population-based birth cohort	2002–2006	The Netherlands
GINIplus	Population based birth cohort	1995–1998	Germany
GLAKU	Population-based birth cohort	1998	Finland
HBCS	Population-based birth cohort	1934–1944	Finland
Health2006	General population study	2006–2008	Denmark
INMA	Population-based birth cohort	1997–2008	Spain
Inter99	Population-based randomized intervention study	1999–2006	Denmark
LISA	population based birth cohort	1997–1999	Germany
MAAS^a^	Population-based birth cohort	1996/1997	UK
MOBA	Population based birth cohort	1999–2008	Norway
MUSP	Pregnancy general population	1981–1984	Australia
NTR^a^	Birth general twin population	From 86—ongoing	Netherlands
NFBC1966 and NFBC1986	longitudinal birth cohort	1966 and 1986	Finland
PIAMA	Population based birth cohort, enriched for high risk allergy children (allergic mother)	1996–1997	Netherlands
Project Viva^a^	Population based birth cohort	1999–2002	USA
Qtwin	Longitudinal twin study	1980–2004	Australia
Raine	Longitudinal pregnancy cohort study	1989–1991	Australia
SKOT	Observational cohort study, monitoring healthy young children from 9 to 36 months of age.	2006–2007 (SKOT I); 2011–2013 (SKOT II)	Denmark
STRIP	Prospective randomized life-style intervention trial	1990–1992	Finland
TCHAD	Birth general twin population	1985–1987	Sweden
TDCOB	Case–control study	Children and adolescence with obesity: 2007–2013; Population-based sample: 2010–2013	Denmark
TEDS	Population based twin birth cohort	From 1994—Ongoing	UK
TRAILS-pop	Population based	2001/2002	Netherlands
TRAILS-CC	High risk	2004	Netherlands
Young Finns	Population based follow-up from childhood to adulthood	1980	Finland

^a^Includes individuals from non-European descent

**Table 3:** Data collected

Cohort	N genotyped children^a^	Phenotypes	Age periods data available				
			Pregnancy	Pre-school	School	Adolescence	Adult
ABCD	1192	Broad	x	x	x	x	
ALSPAC	10,000	Broad	x	x	x	x	x
B58C	6491	Broad	x	x	x	x	x
BAMSE	2500	Broad	x	x	x	x	x
BMDCS	1885	Broad		x	x	x	x
Breathe	1667	Broad			x		
CATSS	13,576	Broad, focus on psychiatry	x, information from registers		x	x	x
CHOP	43,320	Broad		x	x	x	
CHS	3986	Broad, focus on respiratory and metabolic health			x	x	
CLHNS	1779	Broad		x	x	x	x
COPSAC-2000	411	Broad	x	x	x	x	x
COPSAC-2010	700	Broad	x	x	x		
COPSAC-REGISTRY	1240	Broad	x	x			
DNBC -GOYA DNBC -PTB	1500	Broad	x	x	x	x	
	1500						
EFSOCH	812	Anthropometric and glycemic traits	x	x			Parents only
Finntwin12	1264	Broad	Retrospective	Retrospective	x	x	x
Gen3G	582	Broad, focus on metabolic/adiposity	x	on-going			
Generation R	5731	Broad	x	x	x	x	
GINIplus	835	broad	x	x	x	x	Ongoing
GLAKU	357	Broad	x	x	x	x	x
HBCS	1566	Broad		x	x	x	x
Health2006	2802	Cardiovascular disease, type 2 diabetes, and other lifestyle related diseases					x
INMA	1517	Broad	x	x	x	Ongoing	
Inter99	6184	Cardiovascular disease, type 2 diabetes, other lifestyle related diseases, glucose tolerance					x
LISA	674	Broad	x	x	x	x	Ongoing
MAAS	919	asthma and allergy focused	x	x	x	x	Ongoing
MOBA	17,000	Broad	x	x	x	x	x
MUSP	1200	Broad	x	x	x	x	x
NTR	7750	Broad	x	x	x	x	Ongoing
NFBC1966 NFBC1986	5402	Broad	x	x	x	x	x
	3743						
PIAMA	2113	Broad, focus on respiratory health	x	x	x	x	
Project Viva	1580	Broad	x	x	x	x	
Qtwin	4500	Broad			x	x	x
Raine	1500	Broad	x	x	x	x	Ongoing
SKOT I	260	Dietary intake, growth, cognitive development, overweight and lifestyle related diseases		x			
SKOT II	112						
STRIP	666	Broad	x	x	x	x	x
TCHAD	990	Broad			x	x	x
TDCOB	1771	Overweight and Obesity		x	x	x	x
TEDS	10,346	Broad		x	x	x	x
TRAILS-pop	1354	Broad	Retrospective	Retrospective	Retrospective	x	x
TRAILS-CC	341						
Young Finns	2442	Broad	x	x	x	x	x

^a^Some cohorts also have genotype data on parents

Most cohorts were established with the aim of investigating risk and protective factors for a broad range of developmental phenotypes. They have collected data on physical traits, cognition, emotional and behavioral problems, as well as on lifestyle and environmental factors, such as smoking during pregnancy and physical exercise. Other cohorts were set up with a specific focus, such as asthma research, but many of these have collected ancillary information on a wider range of phenotypes. Table 2 gives an indication as to whether data collection was focused on a specific phenotype. Additional details on many of these studies will be available from cohort websites and publications (see Table 1).

Participating cohorts have obtained DNA from blood samples, saliva or buccal swabs. A variety of different genotyping arrays have been used over the years, but meta-analysis has been facilitated by imputation of directly genotyped data using reference panels such as those generated by 1000 Genomes or the Haplotype Reference Consortium [15, 16]. Moreover, an increasing number of cohorts have, or plan to get, additional ‘omics data including parental genotypes, DNA methylation profiles, RNA expression levels, metabolomics and/or microbiome data.

## Results of the genetic studies performed in the EGG and EAGLE consortia

The implementation of GWA meta-analyses for each of the phenotypes of interest to EGG or EAGLE has usually been championed and organized at the level of a working group, formed by a subset of motivated investigators and analysts, who have assumed responsibility for assembling, combining and interpreting the genetic data. The wide range of phenotypes available to study across these consortia has provided fertile ground for many such working groups and has resulted in a large number of peer-reviewed papers across this wide range of phenotypes [17–45]. These are typically GWA meta-analyses, focusing on the effects of individual genetic variants, but increasingly now extend to multivariate, polygenic analyses, that evaluate the joint effects of multiple associated genetic variants and apply this information to address questions of causality.

Amongst the many GWA analyses led by EGG and EAGLE, the traits for which the largest numbers of genetic loci reached genome-wide statistical significance (*p* < 10^−8^) have been birth weight (65 loci), atopic dermatitis (31), childhood BMI (15), allergic sensitization (10), and pubertal growth (10) [17, 19, 23, 26, 28, 36]. For other phenotypes with a large number of genome wide hits, such as age at menarche (108 loci) or ADHD (16 loci), the association analysis has involved collaborations with other consortia [25, 37]. The summary statistics for many of the genome-wide association studies undertaken by EGG and EAGLE investigators can be found on consortium websites (http://egg-consortium.org/; http://www.wikigenes.org/e/art/e/348.html) or are available from corresponding authors.

As with adult phenotype GWA studies, the number of association signals recovered by these studies is influenced heavily by sample size (N = 182,416 for age at menarche, N = 153,781 for birth weight) and, to a lesser extent, by phenotype characteristics (somatic or behavioral traits, continuous or binary outcomes).

In addition to cross-sectional GWA analyses, there have been many examples of projects that have investigated genetic relationships within childhood traits or between childhood traits and related adult phenotypes, often revealing shared genetic factors. For example, genetic overlap was found among related atopic conditions during childhood, and between atopic conditions and auto-immune disorders [19, 36]; among puberty-related phenotypes, and between puberty-related phenotypes and BMI [23, 24, 37]; between childhood and adult blood pressure [41]; between preschool internalizing symptoms and adult psychiatric disorders [18]; and between childhood and adult anthropometric traits [21, 26, 40, 44]. The development of statistical methods that support the calculation of genetic correlations from summary GWAS results [46] and the easy availability of such data from a growing number of GWA meta-analyses for adult traits have enabled these analyses to be undertaken with adequate statistical power.

**Fig. 2:**
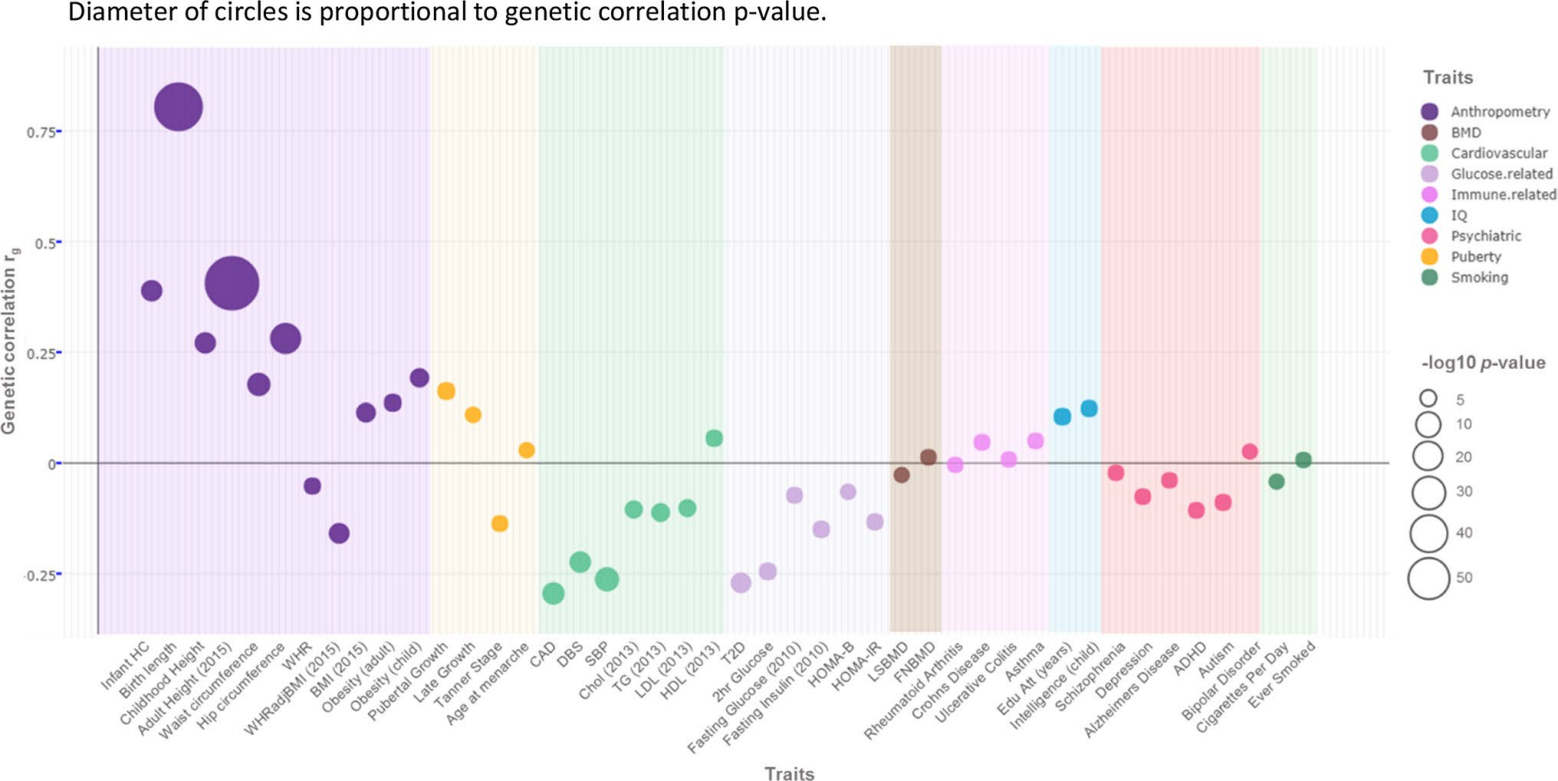
Genome-wide genetic correlation between birth weight and a range of traits and diseases in later life. Genome-wide genetic correlations between birth weight and traits and diseases evaluated in later life. The figure (adapted from Horikoshi et al. 2016 [28] with permission of the authors) displays the genetic correlations between birth weight and a range of traits and diseases in later life as estimated using LD Score regression. Traits selected were those for which genome-wide association summary statistics were available in suitably large sample sizes, and the analyses were typically performed on the largest meta-analyses available as of early 2016. The genetic correlation estimates (r_g_) are colour coded according to phenotypic area. Allelic direction of effect is aligned to increased birth weight. Size of the circle denotes the significance level for the correlation (per the key). Correlations with a lower significance level are not depicted. Further detail on the methods and studies involved is available in Horikoshi et al. 2016 [28]. Diameter of circles is proportional to genetic correlation *p* value

Figure 2 shows genetic correlations, calculated exclusively from GWAS data, between birth weight and a range of continuous and disease phenotypes [28], generated using the linkage disequilibrium score regression approach [46] as implemented in the LDHub web utility [47]. For many cardiometabolic and anthropometric traits measured in late adult life, there is evidence of substantial sharing of genetic variation with birth weight. In line with the wider epidemiological data, the genetic correlations between birth weight and adult cardiometabolic traits (including type 2 diabetes, blood pressure, and coronary artery disease) tend to be negative. These data indicate that a substantial proportion of the observed covariance between birth weight and cardiometabolic disease predisposition is likely to be driven by genetic rather than environmental factors. However, the potential for more complex causal relationships (such as those that connect fetal genotype to adult disease via the correlation with maternal genotype and altered maternal environment) also needs to be considered. Full characterization of these complex relationships requires the application of statistical methods that enable partitioning of genetic effects into maternal and fetal components both at the level of individual SNPs [48] and genome-wide [49]. Using the M-GCTA method [49], for example, it has been reported that maternal genotypes contribute more to gestational weight gain in the mother, while offspring genotypes contribute more to birth weight [45].

Another critical advantage of genetic studies is the potential to characterize causal relationships using Mendelian randomization approaches [50]. Tyrrell et al. [42] found evidence of a positive causal effect of maternal BMI and fasting glucose levels on offspring birth weight but inverse effect of maternal systolic blood pressure on offspring birth weight. Despite bringing together the largest number of studies at the time with relevant data, there was insufficient power to dissect how the opposing effects of maternal glucose and systolic blood pressure are reflected in the maternal BMI effect (one reason why we are keen to extend the collaboration to any new cohorts). Crucially, however, appropriate application and interpretation of studies that seek to elucidate the mechanisms underlying associations between maternal and offspring phenotypes require investigators to consider diverse complicating factors including the correlation between maternal and fetal genetic instruments, and to account for these sources of potential bias in the Mendelian randomization analyses wherever possible [51].

The longitudinal data collected in EGG and EAGLE cohorts provide the means to investigate whether the influence of genetic variants changes over time. This has only recently been explored given the need for large numbers of studies with repeated measures. We have found that genetic variation in FTO, one of the first BMI increasing genetic variants to be identified in GWAS and one of the variants most strongly associated with mean BMI (in adults) is inversely associated with BMI in infancy only becoming positive in later childhood and adult [38], indicating the value of research that explores gene-by-age interactions. On a genome-wide scale, using meta-regression methods, polygenic risk scores generated from adult schizophrenia data yielded associations with variation in childhood and adolescent psychiatric symptom scores, which strengthened in magnitude with increasing age [52].

## Strengths and weaknesses

The aggregation of data in consortia such as EGG and EAGLE provides vastly improved sample sizes and a powerful way to overcome the major weakness of many of the early GWAS, which were, in hindsight, underpowered to detect the generally small genome-wide significant associations. This has brought multiple robust association signals across many traits, and provided a valuable basis for dissecting the, often complex, causal relationships between epidemiologically-correlated traits.

A clear strength of the EGG and EAGLE consortia is the wealth of data available. This encompasses not only repeated measures for physical and behavioral traits, but also copious information on lifestyle and environmental circumstances. Moreover, some of the cohorts have collected data for several decades, and now provide repeated measures well into adulthood. This enables developmental research as well as analyses of the interplay between genes and environment.

To date, one of the limitations has been that the majority of participating cohorts have data based on European-ancestry populations (see Table 2 for exceptions). There is a clear need for equivalent data to be generated in samples from other ethnic groups, so that the genetic contribution to reproducible ethnic differences in the distribution of early life phenotypes can be explored and the implications for adult disease risk quantified.

Since the cohorts are population-based and lack a particular disease-focus, the consortia are not so well-suited to investigate conditions with a low prevalence. They are better-placed to analyze common traits, particularly those that can be measured on continuous scales and analyzed as quantitative measures, such as blood pressure instead of hypertension and ADHD symptom score instead of ADHD diagnosis [32, 34]. Power analyses demonstrate that identification of a genetic variant is, in most circumstances, more powerful for continuous traits than for dichotomous variables based on clinical cut-offs [53].

## Future

Considerable progress is to be expected from ongoing increases in sample sizes, especially for traits such as childhood aggression, ADHD-related traits and internalizing symptoms, where the number of identified genetic variants has been limited so far. Access to new data sets can motivate efforts to tackle phenotypes that have not hitherto been subject to detailed genetic analysis.

The results emerging from many of these studies provide a timely reminder that analysis of early life phenotypes often requires researchers to consider the joint impacts of multiple genomes (e.g., those of the fetus and the mother) together with the web of environmental influences as potential contributors to individual variation. They also highlight the need to take into account the changes happening throughout development. This is now possible because of large, rich and complex datasets that support use of novel statistical methods for the analysis of causality or gene-by-age interaction [48, 49, 51, 54]. There have already been several examples of papers performing such analyses and this will only increase with the number of identified genetic variants. In addition, existing gender differences in the associations between early life and adult factors (such as cardiometabolic risk) suggest a need for more thorough analysis of the effects of gender on these early acting mechanisms. The focus to date on the role of maternal and offspring GWAS information indicates a failure to properly consider the contribution of genetic variation in the father that will be remedied as more data from complete trios and pedigrees becomes available.

We are also planning to expand these consortia to accommodate access to the increasing amount of ‘omics data now becoming more available. Combining the results from EGG and EAGLE GWA analyses with those from DNA methylation analyses performed by the Pregnancy And Childhood Epigenetics (PACE) consortium [55] and with the pregnancy/child cohorts in the COnsortium of METabolomic Studies (COMETS; https://epi.grants.cancer.gov/comtets/) will shed further light on the biological mechanisms underlying associations of early-life risk factors and childhood, adolescent and adult health outcomes.

The focus on translating this knowledge to clinical and public health settings represents a major motivation. Insight into genetic factors underlying stability in traits such as obesity and psychiatric disorders may aid in providing targeted interventions to the groups at highest need. A more complete understanding of the contributions of genetic and non-genetic factors in the relationships between early life and later life traits may focus attention on the most effective strategies for behavioural or environmental modification.
